# Cannabis for female orgasmic disorder/difficulty: a systematic review

**DOI:** 10.1093/sexmed/qfaf061

**Published:** 2025-08-12

**Authors:** Suzanne Mulvehill, Jordan Tishler

**Affiliations:** Female Orgasm Research Institute, Pompano Beach, FL 33062, United States; Association of Cannabinoid Specialists, inhaleMD, Brookline, MA 02445, United States

**Keywords:** female orgasmic disorder, female orgasm difficulty, cannabis, cannabis-assisted orgasm

## Abstract

**Background:**

Cannabis is increasingly recommended to treat female orgasmic disorder/difficulty (FOD/difficulty), a condition that affects up to 41% of women worldwide with no conventional medications.

**Aim:**

To systematically review the existing literature on cannabis and its impact on female orgasm function.

**Methods:**

A systematic review based on the PRISMA model evaluated the effects of cannabis on orgasm function in females with or without FOD/difficulty. Risk of bias was assessed for randomized and nonrandomized studies. Searches were conducted in PubMed, Google Scholar, Cochrane, and Embase.

**Outcomes:**

Primary outcomes focused on the impact of cannabis on female orgasm function.

**Results:**

Sixteen studies met inclusion criteria: 1 randomized controlled trial and 15 observational studies, including data from 8849 females. Most were nonrandomized designs without comparator groups and high risk of bias. Most included both sexes and reported dichotomized outcomes by sex. None excluded females with self-reported orgasm difficulty; 1 controlled for its prevalence; 1 dichotomized females by the presence or absence of orgasm difficulty; and no studies used a clinical diagnosis of FOD. Nine studies investigated cannabis use prior to sexual activity. All 9 studies cited improvements in female orgasm function, including increases in frequency, ease, intensity, quality, and/or multiorgasmic capacity. However, 1 study found cases of situational anorgasmia, and 1 reported that women had more difficulty with focus, potentially leading to orgasm difficulty. Two studies assessed general cannabis use and sexual function: 1 found no association between the frequency of cannabis use and female sexual problems, while the other noted improved orgasm and reduced dysfunction with more frequent use. Five studies examined cannabis alongside other substances, before sex or not: 1 linked inhibited orgasm to combined cannabis and alcohol use, 1 to noncannabis substances, 2 found improved orgasm function with cannabis, and 1 reported improved orgasm function and cases of inability to orgasm due to a lack of focus.

**Clinical Implications:**

Cannabis appears to be a promising treatment option for FOD/difficulty.

**Strengths and Limitations:**

This review found consistent evidence that cannabis improves orgasm function in females with or without FOD/difficulty. Limitations include insufficient high-quality studies and limited reporting on cannabis dosage and timing.

**Conclusion:**

FOD/difficulty should be recognized as a qualifying condition for medical cannabis use. Given the existing evidence supporting its potential efficacy, medical cannabis warrants consideration as a first-line treatment. More randomized controlled trials are needed to clarify optimal dosing, routes of administration, strain specificity, timing of use, and differential effects across FOD subtypes.

## Introduction

This systematic review evaluates the potential efficacy of cannabis in treating clinically diagnosed female orgasmic disorder (FOD) and self-reported female orgasm difficulty. FOD is defined as a marked delay in, infrequency of, or absence of orgasm or markedly reduced intensity of orgasmic sensations, present for at least 6 months and causing significant distress.^1^

While FOD is the formal clinical diagnosis outlined in the *DSM-5-TR*, it is based entirely on a woman’s self-report.^1^ In the literature, this is often referred to more broadly as *female orgasm difficulty*. The key lies in whether a formal clinical diagnosis is present. This distinction is significant, as most patients do not discuss sexual concerns with their health care providers, and many providers do not routinely inquire about them.^2^ Additionally, numerous studies report on female orgasm difficulty vs the clinical diagnosis of FOD.^3-8^ We use the term *FOD/difficulty* to encompass diagnosed and self-reported female orgasm difficulties.

Researchers found that FOD/difficulty poses a substantial public health concern due to its negative impact on women’s health and quality of life.^9^ Women reporting FOD/difficulty, a form of sexual dysfunction, demonstrate higher rates of mental health conditions,^10-13^ including anxiety and depression,^14-16^ posttraumatic stress disorder,^17-20^ and increased use of prescription medications.^21-23^ Increased incidences of childhood sexual abuse have also been identified among women who experience FOD/difficulty,^24^^,^^25^ as well as physical health issues such as cardiovascular disease,^26^^,^^27^ diabetes,^28^ and pelvic floor disorders.^29^ Furthermore, sexual dissatisfaction, including orgasm problems, have been associated with suicidal thoughts among female veterans and military personnel, reflecting the seriousness of the condition.^1^

Despite its high prevalence, treatment options are limited,^30^^,^^31^ innovation in therapeutic approaches is lacking,^31^ and no prescription medications have been approved by the US Food and Drug Administration specifically to treat FOD/difficulty.^32^

### Early observations of cannabis and sexual effects

Although the inclusion of women in National Institutes of Health (NIH)–funded research was not mandated until 1994,^33^ early accounts of cannabis’ effects on sexual experience began surfacing in the literature as early as the 1930s.^34^ In 1934, Dr Walter Bromberg published one of the earliest clinical studies on cannabis use in the *American Journal of Psychiatry*, noting that cannabis could act as a sexual stimulant.^34^ Decades later, Charles Tart’s 1971 book *On Being Stoned* found that cannabis users frequently described sexual orgasm as acquiring “new, pleasurable qualities.”^35^ Similarly, Fisher and Steckler’s 1974 psychological study of cannabis users included participant accounts of enhanced sexual pleasure, even though sexuality was not the study’s primary focus.^36^ Traub’s 1977 research echoed this theme: cannabis users reported increased sexual pleasure, even though the study did not evaluate sexual function directly.^37^

### Early observations of cannabis and orgasm facilitation

While earlier accounts noted enhanced sexual pleasure with cannabis use, sociologist Dr Erich Goode recorded a case in which a woman could achieve orgasm only under the influence of cannabis—an observation he first shared in his 1970 book *The Marijuana Smokers*, based on his 1969 research, and revisited in a 1972 article in *Sexual Behavior*.^38-40^ This was followed by Barbara Lewis, who in *The Sexual Power of Marijuana* (1970) presented narratives from women who reported learning to orgasm with the aid of cannabis—some of whom no longer required it once they had learned—prompting her to suggest that cannabis may have therapeutic potential for treating orgasm difficulty.^41^ Koff also contributed to this emerging field, publishing one of the earliest empirical studies evaluating the relationship between cannabis dosage and sexual response.^42^ His 1974 article included firsthand reports from women describing enhanced orgasmic capacity, including multiple orgasms, as well as cases in which orgasm was inhibited, suggesting a potential dose-dependent effect.^42^

Psychiatrist Helen Singer Kaplan also contributed to this early literature on cannabis and sexual function. In her 1974 book *The New Sex Therapy*, she noted that some individuals reported more prolonged and pleasurable orgasms after cannabis use, including “enhanced orgasmic muscular contractions”—a phenomenon that she emphasized had yet to be investigated under controlled conditions.^43^ Peer-reviewed research by Dawley et al in 1979 reinforced the idea that cannabis may have therapeutic relevance for treating sexual disorders.^44^

### Long-standing research gap

Despite early reports suggesting that cannabis could support orgasmic function in women, systematic research into this topic was not meaningfully pursued. After a 1984 publication by Weller and Halikas^45^ (discussed later), there was a nearly 3-decade gap in empirical research examining the relationship between cannabis and orgasm function. Research interest in this area began to reemerge only in the mid-2000s.

This absence of research occurred despite data showing that up to 41% of women report difficulty reaching orgasm,^46^ an unchanged statistic for 50 years.^47^ Only in recent years has empirical research begun to revisit cannabis’ potential therapeutic role in this context, signaling renewed interest in a question that has lingered in the margins of the literature for over half a century.

### Sex differences in cannabis’s effects on sexual function

Women rate the positive sexual effects of cannabis higher than men.^48^ Gorzalka et al reported that, although research has been limited by the subjective nature of self-reported data, overall findings converge on the positive impact of cannabis on female sexuality in 2 areas: sexual desire and sexual functioning, the latter including sexual satisfaction and orgasmic quality.^48^

In contrast, earlier literature suggested that cannabis use may impair male sexual function, particularly erectile ability.^49^ However, more recent self-report data indicate that most men do not perceive cannabis as detrimental to their sexual performance.^50^ These divergent findings may reflect evolving patterns of use, improved cannabis quality, or methodological differences across studies.

### Rationale for investigating cannabis as a treatment for FOD/difficulty

Cognitive distraction, particularly in the form of automatic negative thoughts, has been consistently linked to lifelong FOD, also known as primary anorgasmia.^51^^,^^52^ Research suggests that Δ^9^-tetrahydrocannabinol (THC), the main active compound in cannabis, may reduce these habitual distractions by inducing a dishabituating effect, a theory first proposed by Feeney^53^ in 1976 and later formalized as the dishabituation theory.^54^ This effect may facilitate orgasm by helping women focus more fully on physical sensations.^54^

Additionally, THC’s interaction with cannabinoid receptors in the prefrontal cortex, an area of the brain linked to inhibition and executive function, has been shown to disrupt activity in this region.^55^ This disruption may contribute to reduced sexual inhibition, a known vulnerability factor in FOD/difficulty.^5^

Sexual trauma and its neurologic impact warrant investigation in this context. Hypervigilance, anxiety, and posttraumatic stress disorder—responses associated with amygdala activity—are known to impair sexual response.^17-20^ THC has been shown to attenuate threat-related amygdala reactivity, particularly in trauma-exposed individuals.^56^ Moreover, cannabis has demonstrated statistically significant improvement in orgasm function among women with a history of sexual abuse.^6^

Other related mechanisms include the role of sexual fantasy and imagination, both of which are linked to improved orgasm function.^57^^,^^58^ Cannabis has been associated with increased sexual fantasy and sensory enhancement, which may support enhanced orgasm function.^35^^,^^59^

The endocannabinoid system (ECS), which modulates arousal, mood, and stress, has been implicated in sexual functioning and presents a promising pharmacologic target.^60^^,^^61^

Finally, female orgasm and cannabis-induced states have been described as altered states of consciousness (ASCs),^62-64^ suggesting the possibility of a shared neuropsychological mechanism.^65^ Notably, one study found that ASCs were positively correlated to higher sexual responsiveness in women.^66^ The ability of cannabis to facilitate such states may support orgasmic function by enabling some women to surrender more fully to bodily sensations.^67^

### Research questions

Given these converging lines of evidence, a systematic review is warranted to evaluate the clinical potential of cannabis in treating women with FOD/difficulty. The primary research question guiding this review is as follows: Does cannabis use improve orgasm function—including orgasm frequency, ease, or satisfaction—for women with FOD/difficulty? The secondary question asks the following: Is there greater orgasm improvement among women who use cannabis in targeted treatment (eg, prior to sexual activity) as compared with those who use it randomly or recreationally?

## Methods

We conducted our review in accordance with the PRISMA guidelines.^68^ To ensure a comprehensive and methodologically sound systematic review, we selected 4 major databases—PubMed, Google Scholar, Cochrane, and Embase—based on their relevance, breadth, and complementary strengths in indexing biomedical and health-related literature. The main keywords used for the search were “cannabis OR marijuana and sex,” “cannabis OR marijuana AND orgasm,” “cannabis OR marijuana AND female sexual function,” “cannabis OR marijuana AND female sexual dysfunction,” “cannabis OR marijuana AND sexuality,” “cannabis or marijuana AND sexual health,” and “cannabis OR marijuana AND sexual function.” The PRISMA framework^69^ was used to capture the data through the different phases of the systematic review, mapping out the number of records identified, included, and excluded and the reasons for exclusions.

### Study selection

Studies were included that evaluated the positive or negative effects of cannabis use on adult females, regardless of whether cannabis was used before sex, and where participants reported its impact on orgasm. The study selection criteria are detailed in turn.

### Diagnostic criteria

The systematic review included studies that enrolled women who (1) self-identified as having orgasm difficulties, (2) were clinically diagnosed with FOD, or (3) participated in cannabis and sex studies that evaluated orgasm function regardless of whether FOD/difficulty was used as an inclusion criterion. Given the high prevalence of orgasm difficulties among women^46^ and the well-documented tendency for such issues to go unreported in clinical settings,^2^ we chose to include studies that assessed orgasm function even when participants were not explicitly recruited according to orgasm-related concerns.

### Inclusion and exclusion criteria

Our eligibility criteria included randomized controlled trials (RCTs) or observational studies that enrolled females ≥18 years of age and evaluated cannabis use and sexual function. Our outcomes of interest were orgasm function and adverse effects. We excluded literature reviews, books, case studies, male-only studies, and studies that focused exclusively on other domains of female sexual functioning (eg, desire, arousal).

### Risk of bias

Risk of bias was assessed by ROBINS-I version 2 (Risk of Bias in Non-randomized Studies–of Interventions) for nonrandomized studies^70^ and the Cochrane Risk of Bias 2 tool for RCTs.^71^

### Procedure

The search was conducted in the aforementioned platforms and electronic databases between April 29 and May 7, 2024, and for new studies that were conducted after May 7, 2024, on January 29, 2025. After compilation of the reviewed list, studies were searched for the term “female orgasm” or “orgasm.” If these terms were not included in the study results, the studies were excluded. When there was uncertainty about whether a study met the treat criteria, 2 reviewers independently reviewed the full text. Disagreements were resolved through discussion and consensus.

### Definition of key outcome measures

Orgasm function was defined as a multidimensional aspect of sexual health encompassing the frequency, ease, satisfaction, or quality or intensity of orgasm. Multiorgasm, defined as the ability to experience >1 orgasm within a single sexual encounter, was also included as a key outcome measure. While difficulty achieving even a single orgasm is central to FOD/difficulty, the presence of multiorgasm may represent an enhanced orgasmic capacity and serve as a therapeutic signal in some women. Specifically, definitions of key outcome measures are as follows:

- Orgasm frequency: how often a woman is able to reach orgasm during sexual activity or stimulation.
- Orgasm ease: how easily or quickly orgasm is achieved, including the level of effort, stimulation, or time required.
- Orgasm satisfaction: the degree to which the orgasm is perceived as pleasurable, fulfilling, or emotionally satisfying.
- Orgasm quality or intensity: the physical and emotional depth of the orgasmic experience, including the strength of muscular contractions and the sensation of release.
- Multiorgasm: the ability to experience >1 orgasm within a single sexual encounter.

### Data extraction and coding of results

The studies that evaluated the nature of cannabis use and its relationship to orgasm function were organized into 3 tables:

- Cannabis use before sexual activity
- General cannabis use and sexual activity
- Cannabis use, before sex or not, in combination with alcohol or other substances and its effect on sexual activity

Each study within the tables was categorized by year of publication, and key data were extracted to allow for sorting and summarization of main findings. The following variables were recorded for each study:

- Authors and year of publication
- Country where the study was conducted
- Sample characteristics: number of participants, gender distribution, and population type (eg, college students, general population, or clinical population)
- Number of female participants
- Study methodology (eg, cross-sectional survey, interview, RCT)
- Reported outcomes related to orgasm function: orgasm frequency, orgasm ease, orgasm satisfaction, orgasm quality/intensity, multiorgasm

For each study, an overall assessment of orgasm function was recorded as *increase*, *no change*, *decrease*, or *mixed*, based on whether cannabis use was associated with reported changes in orgasm-related outcomes. An *increase* rating was applied when ≥1 of the orgasm domains (frequency, ease, satisfaction, quality/intensity, and multiorgasm) showed improvement. *No change* was used when no difference was reported, *decrease* was applied when studies reported inhibited or impaired orgasm and mixed was applied when both orgasm improvement and inhibited or impaired orgasm was were reported. Any nuanced results—such as isolated cases or minor exceptions where the effects varied—were noted in the Notes column of each data extraction table.

All summary judgments were made independently by 2 reviewers and confirmed through discussion to ensure consistency in coding.

## Results

**Figure 1:**
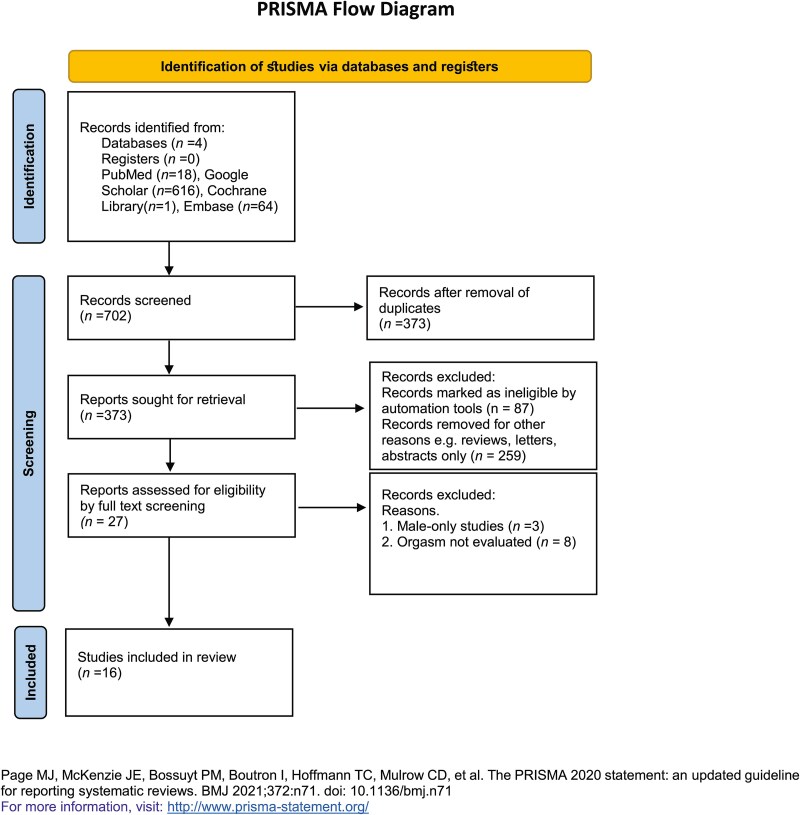
PRISMA flowchart.

A total of 702 peer-reviewed articles were identified through the systematic search. After screening and application of inclusion and exclusion criteria, 686 articles were excluded. The final analysis included 16 studies—1 RCT and 15 observational studies—published between 1974 and 2024, encompassing 15 435 participants, the majority of whom were female (58.8%, n = 8849). Figure 1 shows the study selection process following the PRISMA 2020 guidelines.^68^

Of the 16 studies, 9 examined cannabis use immediately prior to sexual activity, reflecting an intentional approach to alter sexual experience^6^^,^^42^^,^^44^^,^^45^^,^^50^^,^^72-75^ or, as noted in 1 study, to treat orgasm difficulty.^6^ These 9 studies included 2402 participants, 72% of whom were female (n = 1722). Two studies assessed general cannabis use and sexual function,^76^^,^^77^ involving 9102 participants, 52% of whom were female (n = 4751). Five additional studies evaluated the combined effects of cannabis and alcohol or other substances on sexual function, before sex or not,^78-82^ totaling 3931 participants, 60% of whom were female (n = 2376). These results are presented in Tables 1 to 3.

**Table 1:** 

**First author (year); country**	**Sample**	**No. of females** ^**a**^	**Study methodology: design**		**Orgasm frequency**	**Orgasm ease**	**Orgasm satisfaction**	**Orgasm quality/** **intensity**	**Multiorgasm**	**Orgasm function**	**Notes**
Koff (1974)^42^; US	251 adult cannabis users (128 female, 123 male)	128	Self-reported comparison of sexual activity with and without cannabis • Mixed methods (survey and interviews)		Increase				Increase	Mixed	“Our survey reviewed cases of situationally non orgasmic females following marijuana use. There were also cases of multiorgasm” (p 203).
Dawley (1979)^44^; US	84 graduate students; 22% female	18	Assessed attitudes on cannabis use and sexual enjoyment • Survey					Increase		Increase	
Halikas (1982)^72^; US	100 regular marijuana users and 50 nonusers	37	Structured interviews on cannabis use and sexual performance • Interview		Increase			Increase	Increase	Increase	
Weller (1984)^45^; US	97 regular cannabis users by self-report (38% female)	37	Structured interviews assessing cannabis use and sexual behavior • Interview		Increase			Increase	Increase	Increase	
Lynn (2019)^73^; US	373 females; 127 used cannabis before sexual activity; 49 used cannabis but not before sex; 197 did not use cannabis	373	Examined the perceived influence of cannabis on sexual function • Survey				Increase			Increase^*^ (*P* = .04)	
Wiebe (2019)^74^; Canada	216 men and women with experience using cannabis with sex (63% female)	133	Online questionnaire completed by individuals with experience using cannabis during sex • Survey			Increase^*^ (*P* = .039)		Increase		Increase	“We found only a few differences between men and women, with women having more difficulty with focus and less difficulty achieving orgasm when using cannabis” (p 1761).
Moser (2023)^50^; US	811 male and female cannabis users (64.9% female)	526	Examined the influence of cannabis on sexual functioning and satisfaction • Survey					Increase	Increase	Increase	
Mulvehill (2024)^6^; US	387 female cannabis users with and without self-reported orgasm difficulty (n = 202)	387	Assessed the effect of cannabis use before partnered sex on women with and without orgasm difficulty • Survey		Increase^*^ (*P* < .001)	Increase^*^ (*P* < .001)	Increase^*^ (*P* < .001)			Increase	
Banbury (2024)^75^; UK	83 females at least 6 mo after cancer treatment with acquired sexual dysfunctions	83	Assessed the effectiveness of a brief online mindful compassion group intervention adjunct with cannabis suppositories • Randomized controlled trial							Increase^*^ (*P* < .001)	“Sexual function, including arousal, lubrication and orgasms, improved for the mindful compassion group (*P* = .002) and the cannabis suppository / mindful compassion group COCM (*P* < 0.001)” (p 1).
											

Abbreviation: COCM,.

^a^ Total: n = 1722.

^*^Indicates statistically significant results *P* < .05.

**Table 2:** 

**First author (year); country**	**Sample**	**No. of females** ^**b**^	**Study methodology: design**	**Orgasm function**	**Notes**
Smith (2010)^76^; Australia	8650 men and women (4299 women, 49.6%)	4299	Examined the association between cannabis use and a range of sexual health outcomes • Survey	No change	“Orgasm-related problems associated with *daily* cannabis use in men; no associations at any frequency in women” (p 790)
Kasman (2020)^77^; US	452 females, 26% (n = 118) reported sexual dysfunction	452	Assessed the association of cannabis on female sexual function using the Female Sexual Function Index • Survey	Increase^*^ (*P* < .02)	

^a^Note that no studies reported on orgasm frequency, orgasm ease, orgasm satisfaction, orgasm quality/intensity, and multiorgasm. ^b^Total: n = 4751. ^*^Statistically significant improvement was observed in ≥1 of the orgasm-related domains of the Female Sexual Function Index.

**Table 3:** Cannabis use, other substances and sexual function.

**First author (year); country**	**Sample**	**No. of females** ^**b**^	**Study Methodology** **Study Design**	**Orgasm ease**	**Orgasm quality/** **intensity**	**Orgasm function**	**Notes**
Johnson (2004)^79^; US	3004 (60% female)	1801	Assessed the association of sexual dysfunction and comorbid drug and alcohol use by examining inhibited orgasm, functional dyspareunia, inhibited sexual excitement, and inhibited sexual desire. • Survey			Decrease^*^ (*P* = .0001)	“Those who experienced inhibited orgasm (n = 342) were more likely to be female than male, had no high school degree or GED, were unemployed, were in poor health, had a limiting health condition, and reported each of the *DSM-III* psychiatric disorders examined” (p 59).
Sumnall (2006)^82^; UK	270 members of the public aged 18-66 y not seeking drug treatment (48.5% female)	131	Investigated subjective experiences of sex after alcohol or drug intoxication • Survey	Increase^*^ (*P* < .05)		Increase	
Palamar (2018)^78^; US	24 men and women who engaged in sexual intercourse with and without cannabis before sex within the last 3 mo (50% female)	12	Investigation comparing psychosocial and physical sexual experiences related to alcohol and cannabis use • Interview		Increase	Mixed	Some females reported an inability to achieve orgasm on marijuana due to a lack of proper focus.
Roman (2021)^81^; Spain	274 (67.52% female) 18-30 y old, regular cannabis or alcohol users or nonusers	185	Evaluated the influence of cannabis and alcohol on sexuality • Survey			Increase^*^ (*P* < .03)	
Jepsen (2024)^80^; Germany	359 male and female medical students (69% female)	247	The consumption frequencies of illicit drugs, including cannabis was measured, as were alcohol use habits, compulsive sexual behavior, sexual functioning problems, trauma exposure and posttraumatic stress. Sociodemographic data were also collected. • Survey			No change	

^a^Note that no studies reported on orgasm frequency and orgasm satisfaction. ^b^Total: n = 2376. ^*^Statistically significant results: *P* < .05.

None of the studies excluded participants with FOD/difficulty, and none used the diagnostic criteria of FOD as inclusion criteria. The only RCT included participants with acquired female sexual dysfunction (FSD), including acquired orgasm dysfunction, and specified that these participants did not have sexual dysfunction prior to their gynecologic cancer diagnosis.^75^

### Risk of bias

As shown in Table 4, all 16 studies were assessed for risk of bias. The RCT by Banbury et al was rated as having *some concerns*, primarily due to the lack of blinding of outcome assessors.^75^ Of the 15 observational studies, most were rated as having *high* risk of bias, commonly due to confounding from self-selected cannabis use, selection bias from nonrandom sampling methods, and measurement bias from retrospective self-reporting.

**Table 4:** Risk of bias assessment.

**Study: first author (year)**	Bias arising from randomization process	**Bias due to deviations from intended interventions**	**Bias due to missing outcome data**	**Bias in measurement of outcome**	**Bias in selection of reported result**			**Overall risk of bias**
Randomized								
Banbury (2024)^75^	Some concerns	Low	Low	Low	Some concerns			Some concerns
								
	**Bias due to confounding**	**Bias in selection of participants**	**Bias in classification of interventions**	**Bias due to deviations from intended interventions**	**Bias due to missing data**	**Bias in measurement of outcome**	**Bias in selection of the reported result**	**Overall risk of bias**
Nonrandomized								
Koff (1974)^42^	High	Moderate	Moderate	Low	Moderate	High	Moderate	High
Dawley (1979)^44^	High	High	Moderate	Low	Moderate	High	High	High
Halikas (1982)^72^	High	Moderate	Moderate	Low	Moderate	Moderate	Moderate	High
Weller (1984)^45^	High	Moderate	Moderate	Low	Moderate	Moderate	Moderate	High
Palamar (2018)^78^	High	Moderate	Moderate	Low	Low	Moderate	Moderate	High
Lynn (2019)^73^	Moderate	Low	Low	Low	Moderate	Moderate	Moderate	Moderate
Wiebe (2019)^74^	Moderate	Low	Low	Low	Low	Moderate	Moderate	Moderate
Moser (2023)^50^	Moderate	Low	Low	Low	Low	Moderate	Moderate	Moderate
Mulvehill (2024)^6^	High	Low	Low	Low	Low	Low	Low	High
Smith (2010)^76^	Moderate	Low	Low	Low	Low	Moderate	Moderate	Moderate
Kasman (2020)^77^	Moderate	Low	Low	Low	Low	Moderate	Moderate	Moderate
Johnson (2004)^79^	High	Moderate	Moderate	Low	Moderate	Moderate	Moderate	High
Sumnall (2006)^82^	Moderate	Moderate	Moderate	Low	Moderate	Moderate	Moderate	Moderate
Roman (2021)^81^	High	Moderate	Moderate	Low	Moderate	Moderate	Moderate	High
Jepsen (2024)^80^	Moderate	Moderate	Low	Low	Moderate	Moderate	Moderate	Moderate

### Data collection

Data were collected through surveys in 10 of the 16 studies.^6^^,^^44^^,^^50^^,^^73^^,^^74^^,^^76^^,^^77^^,^^80-82^ Koff used a mixed methods approach, combining surveys and interviews.^42^ Three studies used interviews,^45^^,^^72^^,^^78^ with 2 of the 3 studies conducting follow-up interviews with the same participants 2 years later.^45^^,^^72^ Online assessments via the Mentimeter app^83^ were employed in the Banbury et al study,^75^ and Johnson et al relied on archival data collected between 1981 and 1983.^79^

### Study characteristics

Studies employed a range of statistical methods, from descriptive statistics (eg, frequency counts) to multivariable regression models. Earlier studies (1970s-1980s) primarily used descriptive or χ^2^ analyses, while studies published after 2019 more commonly used regression and multivariate modeling approaches (Table 5).

**Table 5:** Cannabis statistical methods used and key variables.

**First author (year)**	**Cannabis use context**	**Statistical methods used**	**Key variables measured**
Koff (1974)^42^	Cannabis use before sex	Descriptive statistics, χ^2^	Sexual desire, enjoyment, satisfaction, partner satisfaction
Dawley (1979)^44^	Cannabis use before sex	Descriptive statistics only	Perceived effects on pleasure, frequency, orgasm intensity, variety
Halikas (1982)^72^	Cannabis use before sex	Descriptive statistics, χ^2^ test	Sexual performance, sensory perception, emotional closeness, partner preference, frequency of substance use with sex
Weller (1984)^45^	Cannabis use before sex	Descriptive statistics, χ^2^ test	Sexual behavior, orgasm quality, desire, emotional/physical closeness, sensory perception, drug use during intercourse
Lynn (2019)^73^	Cannabis use before sex	Descriptive statistics, χ^2^, Fisher exact, 1-way ANOVA, multivariate logistic regression	Sexual satisfaction, sex drive, orgasm quality, lubrication, dyspareunia; frequency and timing of marijuana use
Wiebe (2019)^74^	Cannabis use before sex	Descriptive statistics, χ^2^ test, *t* test for continuous variables, qualitative thematic analysis	Orgasm intensity, satisfaction, lubrication, focus, touch sensitivity, emotional closeness, sexual confidence
Moser (2023)^50^	Cannabis use before sex	Descriptive statistics, independent samples *t* test, 1-way ANOVA, Pearson correlation, multiple linear regression = 2.582, *P* = .006, *R*^2^ = .029	Sexual functioning and satisfaction, sensuality, masturbation, desire, orgasm intensity, form/frequency of cannabis use
Mulvehill (2024)^6^	Cannabis use before sex	Descriptive statistics, McNemar test, 1-sample *t* test, 1-factor ANOVA	FSFI orgasm subscale; cannabis use frequency, duration, and method; mental health; sexual abuse history
Banbury (2024)^75^	Cannabis use before sex	Repeated measures ANOVA, MANOVA, MANCOVA, post hoc test, content analysis	Sexual function, pain, arousal, lubrication, orgasm, satisfaction, well-being, quality of life
Smith (2010)^76^	Cannabis use in general	Descriptive statistics, contingency tables, logistic regression, multinomial logistic regression, design-based *F* statistics	Cannabis use frequency, number of sexual partners, condom use at last intercourse, STI diagnosis, sexual problems
Kasman (2020)^77^	Cannabis use in general	Descriptive statistics, χ^2^, Fisher exact, *t* test, Wilcoxon rank sum, multivariable linear and logistic regression	FSFI total and subscale scores, cannabis use frequency, method of consumption, chemovar type, demographics, comorbidities
Johnson (2004)^79^	Cannabis use with other substances (alcohol, illicit drugs)	Descriptive statistics, bivariate and multivariate logistic regression	*DSM-III* sexual dysfunctions; lifetime use of marijuana, alcohol, illicit drugs; psychiatric diagnoses; health conditions
Sumnall (2006)^82^	Cannabis use with other substances (alcohol, ecstasy, cocaine, etc)	Descriptive statistics, χ^2^ test, Spearman correlations, Wilcoxon signed rank test, log-linear analysis, backward stepwise linear and logistic regression	Subjective sexual experience, drug use history, sexual activity type and setting, psychometric measures
Palamar (2018)^78^	Cannabis use with other substances (alcohol, illicit drugs)	No inferential statistics; thematic analysis per Atlas.ti, coded independently by 2 raters	Psychosocial and physical sexual experiences
Roman (2021)^81^	Cannabis use with other substances (alcohol)	Descriptive statistics, Kolmogorov-Smirnov test for normality, Student *t* test, 1-way ANOVA, Mann-Whitney *U*, Kruskal-Wallis	Sexual function, cannabis use risk, alcohol use risk, demographics
Jepsen (2024)^80^	Cannabis use with other substances (alcohol, MDMA, etc)	Descriptive statistics, *t* test, *U* test, Pearson and Spearman correlations, stepwise multiple linear regression, ordinal regression, path analysis	CSB, SFP, alcohol use, drug use, trauma, posttraumatic symptoms, substance consumption frequency

Abbreviations: ANOVA, analysis of variance; CSB, compulsive sexual behavior; FSFI, Female Sexual Function Index; MANOVA, multivariate analysis of variance; MANCOVA, multivariate analysis of covariance; SFP, sexual functioning problems; STI, sexually transmitted infection.

### Effectiveness outcomes

All studies evaluated participants on their cannabis use and orgasm function. Four studies specifically addressed FOD/difficulty,^6^^,^^74-76^ and 1 study controlled for the high percentage of women with orgasm difficulty when reporting results.^45^ Kasman et al used the Female Sexual Function Index^84^ to assess sexual function/dysfunction, including FOD/difficulty.^77^ Mulvehill and Tishler used the orgasm subscale questions of the Female Sexual Function Index^84^ with and without cannabis before partnered sex and dichotomized women with and without self-reported FOD/difficulty.^6^ Another study directly asked participants if they had FOD/difficulty.^74^ The fourth study evaluated acquired sexual dysfunction, including acquired FOD/difficulty.^75^

Four studies evaluated cannabis and sexual function in females only.^6^^,^^73^^,^^75^^,^^76^ Of these, 3 focused on cannabis use before sexual activity,^6^^,^^73^^,^^75^ while 1 assessed general cannabis use.^76^

The remaining 12 studies included male and female participants. Among these, 6 assessed cannabis use prior to sexual activity;^42^^,^^44^^,^^45^^,^^50^^,^^72^^,^^74^ 1 examined general cannabis use^77^; and 5 explored cannabis use, before sex or in general, in combination with alcohol or other substances.^78-82^

The majority of studies (62.5%, 10/16) were conducted in the United States.^6^^,^^42^^,^^44^^,^^45^^,^^50^^,^^72^^,^^73^^,^^76^^,^^78^^,^^79^ The remaining 6 studies (37.5%) were conducted in Canada,^74^ Germany,^80^ Spain,^81^ the United Kingdom,^75^^,^^82^ or Australia.^77^ Notably, no studies were published between 1984 and 2003, either in the United States or internationally.

### Orgasm function

Improved orgasm function—including increased frequency, intensity, quality, ease, satisfaction, and the ability to experience multiple orgasms per sexual encounter—was reported in all 9 studies that evaluated cannabis use before sexual activity (Table 1). The RCT that investigated acquired FSD, including acquired FOD/difficulty in patients with gynecologic cancer, revealed statistically significant improvements in orgasm function with cannabis suppositories and mindful use (*P* < .0001).^75^ Another cited statistical significance for improvements in orgasm function—specifically, improvements in orgasm, orgasm satisfaction, and overall sexual experience (*P* < .04).^73^

Of the 2 studies that examined general cannabis use (Table 2), 1 reported increased orgasm function, noting that with each increase in cannabis use frequency, FSD, including orgasm dysfunction, declined by 21%.^76^ The other study found no association between cannabis use frequency and orgasm difficulty or other sexual problems in women, while finding a significant association with orgasm-related problems in men.^77^

In the 5 studies that examined cannabis use alongside other substances before sex or not (Table 3); 2 reported increased orgasm function^81^^,^^82^; 1 noted decreased orgasm function^79^; 1 indicated mixed orgasm response^78^; and 1 found no change with the use of cannabis but a decreased ability to enjoy or reach orgasm associated with the use of other substances (ie, not cannabis), such as benzodiazepines and stimulants (eg, speed).^80^ The study that revealed decreased orgasm function found that inhibited orgasm was significantly associated with marijuana and alcohol use (*P* = .0001).^79^ In this study, participants who experienced inhibited orgasm were more likely to be female, have no high school degree or GED, be unemployed, be in poor health, have a limiting health condition, and meet the criteria for each of the *DSM-III* psychiatric disorders examined. In the study that reported mixed results, there were cases of participants, male and female, reporting “magnified” orgasms, while some women also reported cases of an inability to orgasm due to a lack of focus.^78^

### Orgasm frequency

Across all 3 study groups, orgasm frequency was improved in 3 studies involving cannabis use prior to sexual activity.^6^^,^^45^^,^^72^ Mulvehill and Tishler cited statistically significant improvements in orgasm frequency, with 72.8% of women who reported orgasm difficulty showing increased orgasm frequency (*P* < .001).^6^ Halikas et al found that the number of orgasms increased or variably increased for 27% of the women.^72^ In follow-up interviews conducted 2 years later, 16% of women indicated an increase in the number of orgasms.^45^

### Orgasm ease

Four studies across all 3 study groups demonstrated statistical significance for cannabis improving orgasmic ability.^6^^,^^50^^,^^74^^,^^82^ Sumnall et al cited a statistically significant improvement in the ease of orgasm when comparing cannabis with alcohol and other substances (*P* < .05).^82^ Wiebe and Just reported that 50.8% of women (86/195) experienced an increased ability to orgasm with cannabis use, and half of those who previously indicated orgasm difficulty said that it was easier to reach orgasm while using cannabis.^74^ Moser et al revealed statistically significant improvements in orgasmic ability for men and women associated with cannabis use (*P* < .05).^50^ Mulvehill and Tishler found that 71% of women with orgasm difficulty (143/202) had improved orgasm ease when using cannabis before partnered sex (*P* < .001).^6^

### Orgasm satisfaction

Two studies reported improved orgasm satisfaction when using cannabis before sex,^6^^,^^73^ and 1 study reported improved satisfaction without stating that it was specifically related to orgasm.^82^ Mulvehill and Tishler cited statistical significance for improved orgasm satisfaction for women with and without FOD/difficulty (*P* < .001).^6^ Lynn et al found that women who used cannabis before sex had 2.13-higher odds of reporting orgasm satisfaction, adding that satisfying orgasm was the only statistically significant sexual function domain between those who use cannabis before sex and those who do not (*P* < .04).^73^ Sumnall et al noted that participants who used cannabis or other substances before sex were more likely to report greater sexual pleasure and satisfaction.^82^

### Orgasm intensity and quality

Improved orgasm intensity or quality was reported in 6 studies,^44^^,^^45^^,^^50^^,^^72^^,^^74^^,^^78^ all of which involved cannabis use before sex, with 1 of the studies^44^ also evaluating the separate effects of alcohol on orgasm experiences. Dawley et al described cannabis as increasing orgasm intensity.^44^ Halikas et al found that 60% of women reported enhanced or variably enhanced orgasm quality after cannabis use, controlling for the high proportion of women who rarely or occasionally experience orgasm (*P* = .02).^72^ Weller and Halikas cited a statistically significant enhancement in female orgasm quality associated with cannabis use (*P* = .025).^45^

Palamar et al noted that cannabis appeared to influence the length and intensity of sexual activity and orgasm.^78^ Wiebe and Just found that 65.7% of participants (women and men) reported increased orgasm intensity.^74^ Moser et al found that men and women perceived cannabis use as enhancing sexual functioning and satisfaction, particularly desire and orgasm intensity.^50^

### Multiorgasm

Multiorgasm, also referred to in the research as repeated orgasms per sexual encounter, was noted in 4 studies, all of which used cannabis before sexual activity.^42^^,^^45^^,^^50^^,^^72^ Koff reported cases of women experiencing multiple orgasms per sexual encounter when using cannabis.^42^ Halikas et al noted that that the ability to repeat orgasm increased or variably increased for 8% of the women.^72^ Weller and Halikas reported an increased number of orgasms and the ability to repeat orgasms but not frequently.^45^ Moser et al noted that there was support for orgasm frequency among women, with >40% of women (n = 356) reporting an increased ability to have >1 orgasm per sexual encounter.^50^

### How orgasm was experienced

The majority of studies did not specify how orgasm was experienced (11/16). Of the 5 studies that did report how orgasm was experienced, 3 reported that orgasm was experienced during intercourse,^42^^,^^72^^,^^75^ 1 indicated that orgasm was experienced during partnered sex,^6^ and 1 noted that orgasm was experienced during a sexual encounter while stating that cannabis increases pleasure during masturbation.^50^ One additional study, which did not report how orgasm was experienced, stated that cannabis increased pleasure during oral sex and intercourse.^44^

### Orgasm-related adverse effects and broader sexual impacts

Of the 16 studies reviewed, 8 did not report any cannabis-specific adverse effects related to women’s orgasm, sexual function, or other factors.^44^^,^^45^^,^^50^^,^^72^^,^^73^^,^^75-77^ Notably, Smith et al reported statistically significant adverse effects on men’s orgasm function but found no negative effects on women’s sexual function.^76^

Three studies reported cannabis-specific adverse sexual effects, primarily affecting orgasm or overall sexual enjoyment.^6^^,^^42^^,^^74^ Koff observed situational nonorgasmic outcomes following cannabis use.^42^ Wiebe and Just found that 4.7% of participants stated that sex was worse under cannabis use, linked to difficulty focusing, sleepiness, and distraction, which interfered with the sexual experience, though not directly affecting orgasm.^74^ Mulvehill and Tishler found that 4% of women experienced orgasm difficulty when using cannabis before partnered sex.^6^

Five studies reported adverse effects related to alcohol and drug use.^78-82^ One attributed the adverse effects to cannabis;^78^ 1 identified adverse effects from cannabis and alcohol;^79^ 1 isolated the adverse effects to substances besides cannabis^80^; 2 attributed adverse effects to drug use, including alcohol and cannabis, without specifically isolating cannabis as the specific cause.^81^^,^^82^

Palamar et al reported that some females were unable to orgasm while using cannabis due to a lack of focus.^78^ Johnson et al found statistically significant inhibited orgasm associated with concurrent cannabis and alcohol use (*P* = .0001).^79^ Jepsen et al associated the frequencies of substances besides cannabis—namely, benzodiazepine use (*P* < .001) and cocaine/crack use (*P* = .03)—with a decreased ability to enjoy orgasm.^80^ Roman et al cited 4% sexual dysfunction without isolating cannabis as a contributing factor,^81^ and Sumnall et al associated drug use, including alcohol and cannabis, to poor decision making that could lead to sexual activity that may be considered inappropriate, specifically in instances where explicit consent is required for sex.^82^

### Frequency of cannabis use and orgasm response

Frequency of cannabis use—also referred to as “regular cannabis use,” “experienced cannabis users,” “users,” or “frequency of use”—correlated to increased orgasm function in the majority of studies (9/16) across all 3 study groups.^6^^,^^45^^,^^50^^,^^72-74^^,^^76^^,^^81^^,^^82^ Additionally, 1 study found that the frequency of cannabis use was unrelated to sexual problems with women yet clearly interfered with orgasm in men.^77^

Weller and Halikas evaluated the same participants 2 years after the original Halikas et al study and reported that regular cannabis users, who had used cannabis over 2 years and at least 50 times in the 6 months preceding the initial study, experienced enhanced orgasm quality and an improved ability to experience multiple orgasms per sexual encounter.^45^^,^^72^ Sumnall et al stated that although their findings may primarily apply to heavier nondependent substance users, including cannabis users, orgasm experience and sexual satisfaction were significantly enhanced after using substances, including cannabis, as compared with alcohol.^82^ Lynn et al found that women with frequent cannabis use, regardless of before sex or not, had 2.10-times higher odds of reporting satisfactory orgasms than those with infrequent use.^73^ Wiebe and Just noted that the largest group of cannabis users with experience using cannabis before sex used cannabis daily (37.8%), with 65.7% reporting increased intensity of orgasms.^74^ Kasman et al cited statistical significance for cannabis use frequency and improved orgasm function (*P* = .002).^77^ Roman et al reported that regular cannabis users were recruited and that the relationship between cannabis use and sexual function was statistically significant for orgasm (*P* = .03).^81^

Moser et al reported that the majority of participants (62.8%) used cannabis daily, with 70% reporting that cannabis increased orgasm intensity.^50^ Mulvehill and Tishler cited statistical significance for frequency of cannabis use before partnered sexual activity resulting in a more positive orgasm response for women with or without FOD/difficulty (*P* < .001).^6^

### Dose and orgasm response

Seven studies mentioned dose or the amount of cannabis used.^42^^,^^44^^,^^74-76^^,^^78^^,^^81^ Two studies specifically evaluated dosage,^42^^,^^75^ 3 studies identified dose as being important,^42^^,^^44^^,^^78^ and 1 study evaluated participants who were at high risk of having cannabis-related problems.^81^

Koff reported that the average dose was up to 1 joint for 22.6% of the females and that the THC content ranged from 0.8% to 1.4%, with an average of 1%.^42^ Koff further stated that moderate dosage released inhibitions and that it was evident that as dosage increased, sexual desire decreased.^42^ Dawley et al observed that cannabis’ effects on sexual functioning may be dose related.^44^ Palamar et al noted that dose appears to be an important factor and that too much cannabis use tended to create anxiety, especially in females, and that too much cannabis, for males and females, had an adverse effect on their mindsets during sex.^78^

Kasman et al found a dose-response relationship between increased frequency of cannabis use and reduced odds of FSD.^77^ Wiebe and Just reported that enhanced focus or increased distraction may relate to the amount of cannabis used or individual reactions to cannabis.^74^ Roman et al found that participants who were at high risk of having cannabis-related problems had better sexual functioning, including arousal and orgasm.^81^ Banbury et al noted that dose via cannabis suppositories varied from 100 to 1000 mg, with the largest group reporting 1000 mg (19.3%).^75^

### Cannabis intake methods and orgasm response

Seven studies evaluated intake methods, with all 7 reporting smoking as the primary route.^6^^,^^42^^,^^44^^,^^50^^,^^73^^,^^76^^,^^78^ One study evaluated cannabis suppositories,^75^ and 1 examined cannabis use via smoking joints.^82^ Dawley et al evaluated cannabis smokers only, making no mention of other intake methods, and reported improved orgasm intensity.^44^ Koff stated that 79.8% of females smoked cannabis while 20.1% chose edibles.^42^ Palamar et al published participants’ comments of the effects of smoking cannabis, with positive and negative effects on orgasm function.^78^ Lynn et al found that the majority of users were cannabis smokers and that orgasm satisfaction improved overall.^73^

Kasman et al reported that, as compared with less frequent users, more frequent users tended to smoke flower (49.2%) and have improved orgasm response.^77^ In the Moser et al study, 95.5% of participants reported using cannabis in the form of flower, finding that forms of flower and wax predicted increased sexual functioning and satisfaction.^50^ Mulvehill and Tishler stated that the majority of women with FOD/difficulty smoked cannabis (50%) and the second-largest group used edibles (23.8%), reporting that intake method was statistically significant in relation to a more positive orgasm response for women with and without FOD/difficulty (*P* < .0001).^6^ Banbury et al found that among participants using cannabis suppositories, 44.6% had obtained a formal prescription for cannabis flower but reformulated it themselves into suppositories. When used mindfully, suppositories resulted in a statistically significant improvement in orgasm function (*P* < .0001).^75^ Sumnall et al found that cannabis users smoked a mean 14.8 (SD, 10.5) joints per week and that those who used cannabis to enhance sex were more likely to report positive sexual experiences.^82^

### Reasons for cannabis use and orgasm response

Four studies examined reasons for cannabis use: 3 evaluated participants who used cannabis before sex,^6^^,^^50^^,^^75^ and 1 evaluated participants who used cannabis more generally.^76^ Moser et al found that cannabis was statistically significant for enhancing relaxation during sex, while not evaluating reason of use directly (*P* < .05).^50^ Mulvehill and Tishler reported that 63% of women with FOD/difficulty stated that their main reason for cannabis use was relaxation, with 10.4% mainly using cannabis for sexual activity.^6^ Reason for use was statistically significant and resulted in an improved orgasm response for all women, with and without FOD (*P* < .001).^6^ Banbury et al identified that the main reason why patients with gynecologic cancer and acquired FOD/FSD reported use of cannabis suppositories was to relieve sexual pain during intercourse.^75^ Kasman et al found that the most common reason for cannabis use was relaxation, with 81% citing this as the primary motivation, while reporting that frequency of use contributed to a more positive orgasm and sexual function response.^77^

### Timing of cannabis use

Four studies mentioned timing of cannabis use, with all 4 evaluating participants who used cannabis before sexual activity.^42^^,^^44^^,^^73^^,^^75^ One study examined timing of cannabis use and stated that 24.1% of patients with gynecologic cancer and acquired FOD/difficulty inserted the cannabis suppositories vaginally 30 minutes before sexual intercourse while 21.7% did so 30 minutes to 1 hour before sexual intercourse.^75^ One study mentioned that timing of cannabis use in relation to sex was not defined.^73^ One study reported that participants used different types of cannabis at different times,^42^ and 1 study theorized that cannabis use at the time of a sexual encounter was an individual’s attempt to cope with the stress of the situation.^44^

### Percentage of women who use cannabis and reported lifelong FOD/difficulty

One study reported that 4% of study participants who used cannabis before sex had not yet experienced an orgasm, a condition referred to as primary anorgasmia or diagnostically as lifelong FOD.^6^ Similarly, Roman et al evaluated cannabis and alcohol use and found that only 4% of the study participants indicated sexual dysfunction, while not specifically noting the domain of sexual function, while the remaining 96% reported none.^81^

### Cannabis suggested for treatment or further evaluation for FOD/difficulty and other sexual disorders

Seven studies suggested that cannabis may be a treatment for FOD/difficulty and other male and female sexual dysfunctions.^6^^,^^44^^,^^45^^,^^50^^,^^73^^,^^74^^,^^76^ One study suggested that further research is needed, as it found that sexual function was better in young individuals who used cannabis (or alcohol) more frequently as compared with those who did not use either substance.^81^

Dawley et al reported that there may be value in researching the use of cannabis in the treatment of sexual disorders.^44^ Weller and Halikas recommended that further work is needed to determine if the effects of cannabis on sexual behavior in their study are seen in the broader populations.^45^ Lynn et al wrote that a better understanding of the role of the ECS in women could help lead to development of treatments for FSD.^73^ Wiebe and Just stated that further research is needed to delineate the different effects of cannabis on sexual experience and specifically on sexual dysfunction.^74^ Kasman et al noted that although certain aspects of sexuality were not assessed, such as vaginismus, they represent important areas for further research.^77^ The authors also identified that the ECS represents a viable therapeutic target through cannabis for FSD, warranting future prospective studies.

Moser et al reported that the medical implications of their study include the use of cannabis for treating sexual dysfunctions, especially for women.^50^ Mulvehill and Tishler concluded that their findings support 50 years of speculation and research suggesting cannabis as a treatment for FOD/difficulty.^6^ The key results—that is, statistically significant improvements in orgasm frequency, ease, and satisfaction among women reporting FOD/difficulty during partnered sex—highlight the potential of cannabis becoming a viable therapeutic option.^6^ Banbury et al stated that their findings provide a foundation for future research to develop diverse health care approaches that improve mental health and quality of life for women experiencing FSD following gynecologic cancer treatment.^75^

## Discussion

Consistent reports of improved orgasm function in women with and without FOD/difficulty span 50 years of research, with cannabis suggested as a treatment for sexual disorders since 1979.^44^ Notably, Lewis was the first to explicitly suggest cannabis as a therapeutic aid for FOD/difficulty, then commonly referred to as “frigidity.”^41^ Her book included chapters titled “Marijuana and Frigidity” and “Marijuana as Therapy,” and on page 71 she asked, “Could marijuana be the catalyst that would enable a so-called non-orgasmic woman to reach orgasm?” More than 50 years later, scientific research is beginning to answer that question, not only through systematic investigation but through policy shifts as well, with 2 US states now recognizing cannabis as a treatment for FOD/difficulty.

Additionally, as shown by Lynn et al, women who used cannabis before sexual activity had 2.13-times higher odds of reporting satisfactory orgasms as compared with nonusers.^73^ Furthermore, the observed relationship between cannabis use before sexual activity and frequency of cannabis use improved women’s orgasm function.

### Orgasm improvement in all 3 study groups

Orgasm function was enhanced in all 3 study groups (Tables 1–3), across a range of study designs, populations, and cannabis use contexts, underscoring the consistency of this effect despite methodological differences. Specifically, 4 studies showed significant benefit for women who had FOD/difficulty: 3 demonstrated this in the “cannabis before sexual activity” group^6^^,^^74^^,^^75^ and 1 in the general cannabis use group.^76^

### Women treating their FOD/difficulty with cannabis

The results of 4 studies reveal that women with FOD/difficulty appear to be using cannabis to treat it.^6^^,^^74–76^ Kasman et al noted that 26% of the women in their study reported FSD, including FOD/difficulty, and a more positive orgasm response was statistically significant when evaluating women who used cannabis >3 times per week as compared with <3 times per week.^77^

Mulvehill and Tishler reported that while 53% of the women in their study had FOD/difficulty, the largest group stated that it “almost always or always” orgasmed when it used cannabis before partnered sexual activity, as compared with “almost never or never” when not using cannabis before partnered sexual activity.^6^ Additionally, Mulvehill and Tishler found that 4% of women with FOD/difficulty who used cannabis had primary anorgasmia, meaning that they had not yet had an orgasm despite adequate stimulation. This percentage compares to the average of 10% to 15% of women who report primary anorgasmia.^85^

### Why cannabis improves female orgasm function and FOD/difficulty

To evaluate why cannabis enhances orgasm function in women, particularly those with FOD/difficulty, we aim to (1) correlate the primary psychological factors associated with FOD/difficulty to research on THC’s effects on specific brain regions and (2) review the impact of THC on the ECS.

#### Lack of erotic thoughts, imagination, and fantasy and THC

A lack of erotic thoughts during sexual activity is the strongest predictor of sexual difficulties in women and is especially prevalent among those with FOD/difficulty.^51^ Imaginative erotic stimulation has been shown to improve sexual function,^86^^,^^87^ and greater sexual fantasizing is positively correlated with enhanced orgasm function.^88^ Cannabis has consistently been linked to increased sexual fantasizing,^35^^,^^41^^,^^42^^,^^59^ with early observations by Tart describing marked enhancement of sexual fantasy and sensory imagery.^35^ Together, these findings suggest that cannabis, particularly through the action of THC, may support orgasmic function in part by facilitating erotic imagination and mental arousal in women who struggle to access it naturally.

#### ECS and female sexual functioning

A growing body of research suggests that the ECS plays a key role in regulating trauma- and stress-related disorders.^89^ Lutz highlighted the ECS as a promising target for pharmacologic interventions in mood-related conditions, such as anxiety, posttraumatic stress disorder, phobias, and depression.^90^

Emerging evidence points to the ECS as a potential modulator of female sexual functioning.^88^ Studies have found a relationship between endocannabinoid concentrations and sexual arousal, suggesting that the ECS is involved in male and female sexual processes.^61^ Researchers propose that endocannabinoids may play an important role in the sexual response cycle, offering therapeutic potential for the treatment of sexual disorders.^48^^,^^91^ This connection was supported by several studies in this systematic review.^50^^,^^73^^,^^76^

#### Female orgasm, female orgasm disorder, ASCs, and cannabis

The Second International Consultation on Sexual Medicine, held in Paris in July 2003, assembled >200 multidisciplinary experts from 60 countries and formally defined the female orgasm as “a variable, and transient peak sensation of intense pleasure, creating an altered state of consciousness.”^92^^(p785)^ This definition, grounded in international clinical consensus, marked a significant shift in the understanding of orgasm beyond its physiologic features. Costa et al found that ASCs involving attentional absorption were strongly associated with sexual responsiveness in women and to a lesser degree in men.^66^

Several sources, including empirical research and theoretical analyses, have described the female orgasm itself as an ASC. Davidson noted that the mental state occurring during orgasm is often described as an altered state, noting that it represents “a qualitative departure from usual experience.”^65^ Additional research has found that women frequently report ASC-like experiences during orgasm^62^^,^^64^^,^^65^^,^^93^^,^^94^ and that in some individuals, orgasms can be prolonged; in women, multiple orgasms can also occur in rapid succession, stretching over a relatively long period.^65^

THC, the primary component in cannabis, is known to induce ASCs.^59^^,^^95^ Tart observed that THC intensifies ordinary sensory perception and alters the experience of time.^63^ Mishara and Schwartz proposed that deliberately entering ASCs can have therapeutic effects.^96^

Given that the female orgasm and the cannabis experience are associated with altered states, Mulvehill and Tishler proposed that women who use cannabis regularly may be more adept at accessing these states, allowing for greater surrender to bodily sensation.^67^ They suggested that cannabis-induced ASCs may enhance orgasmic ability. The ASC theory posits that women who learn to intentionally access altered states with cannabis are more likely to experience orgasm.^67^

The concept that altered states can facilitate erotic receptivity is echoed not only in clinical and neuropsychological literature but also in ancient ritual imagery. The frescoes in the *Villa of the Mysteries* in Pompeii, Italy, are widely interpreted as depicting an initiation ritual believed to facilitate transformation through ecstatic states and surrender to the body. Often interpreted as part of a woman’s transition into marriage, the scenes appear to portray a symbolic psychosexual awakening in which the initiate is taught—or learns—to let go, a capacity considered important for facilitating orgasm in women. While not empirical evidence, such imagery suggests a long-standing cultural recognition that altered states may be necessary to access the full erotic capacity of the body. These scenes provide a compelling historical parallel to contemporary theories linking cannabis, altered states, and the facilitation of female orgasm.

#### Cannabis dosage and sexual functioning

Dosage is a critical factor in the relationship between cannabis use and sexual functioning, including orgasm. As early as 1969, dosage was noted as being important for experiencing cannabis’ sexual enhancements.^38^ Lewis suggested that in some cases, dosage may have contributed to inhibited orgasm,^41^ which was identified in 3 studies in this review.^42^^,^^74^^,^^78^ Koff found that sexual enjoyment was more common with low to moderate doses.^42^ Similarly, Balon described a bidirectional effect, noting that while low and acute doses may enhance sexual functioning, chronic use of higher doses may lead to negative effects.^97^ Kipping and Lynn found that moderate doses improved female sexual function—including orgasm, libido, and arousal—whereas higher doses were associated with negative outcomes.^60^ Several studies have reinforced the dose-dependent nature of cannabis’ sexual effects, with inhibiting and enhancing outcomes, often influenced by the amount consumed.^42^^,^^74^^,^^78^ Taken together, these findings underscore the dose-dependent nature of cannabis’ sexual effects, with lower to moderate doses generally associated with enhancement and higher doses more likely to inhibit sexual functioning.

### Limitations

While this review provides valuable insights into the relationship between cannabis use and orgasmic function in women, several limitations should be considered. Most studies relied on self-selected samples, which may not be representative of the broader population. Despite the majority improvements in orgasm-related outcomes, 1 study explicitly noted that cannabis did not help all women orgasm,^6^ and 3 reported mixed results, potentially influenced by dosage or individual variation.^42^^,^^74^^,^^78^

Heterogeneity across studies—including differences in data collection methods, participant demographics, cannabis dosage and intake methods, and outcome measures—limits the precision of direct comparisons. Additionally, outcome reporting formats varied: some studies conducted statistical analyses, others relied solely on descriptive findings, and several used both. This inconsistency may have introduced reporting bias and complicates the ability to draw firm conclusions across studies.

## Conclusions

Cannabis appears to be a promising treatment for FOD/difficulty, with the majority of studies reviewed reporting improvements in orgasm function and satisfaction among women who use cannabis. These benefits were observed across diverse study designs, populations, and cannabis use contexts. Given this growing body of evidence, FOD/difficulty should be considered a qualifying condition for medical cannabis, and medical cannabis should be evaluated as a potential first-line treatment. These findings suggest a strong association between cannabis use and improved orgasm function, but further RCTs are needed to establish causality and better define key parameters, such as dosage, route of administration, timing of use, strain specificity, and the differential effects on FOD subtypes.
